# Advancing homebrew AI in diagnostic practice: opportunities and barriers^†^

**DOI:** 10.1002/path.6483

**Published:** 2025-09-26

**Authors:** Zaid H Khoury, Ahmed S Sultan

**Affiliations:** ^1^ Department of Oral Diagnostic Sciences & Research School of Dentistry, Meharry Medical College Nashville TN USA; ^2^ Division of Artificial Intelligence Research University of Maryland School of Dentistry Baltimore MD USA; ^3^ Department of Oncology and Diagnostic Sciences University of Maryland School of Dentistry Baltimore MD USA; ^4^ University of Maryland Marlene and Stewart Greenebaum Comprehensive Cancer Center Baltimore MD USA

**Keywords:** laboratory diagnostics, homebrew AI, digital pathology, regulatory frameworks, artificial intelligence

## Abstract

In a recent issue of *The Journal of Pathology*, Calderaro *et al* present a timely and persuasive argument advocating for the integration of homebrew artificial intelligence (AI) models in diagnostic pathology. Their article is a robust defense of local model development within pathology departments as a pathway to democratizing digital diagnostics. This commentary expands on their premise, critically examining the real‐world implications, practical limitations, and unmet needs of practicing pathologists. The commentary outlines both the opportunities and challenges for the widespread adoption of homebrew AI in pathology practice. Without institutional backing, digital infrastructure, and sustained training efforts, the promise of homebrew AI may falter. © 2025 The Author(s). *The Journal of Pathology* published by John Wiley & Sons Ltd on behalf of The Pathological Society of Great Britain and Ireland.

## The case for homebrew AI in diagnostic pathology

As digital pathology continues to advance, many practicing pathologists now recognize the value of digital workflows. Studies have shown that interpretations of scanned histopathological slides are comparable to those made using traditional light microscopy [1]. Moreover, pathology is inherently visual and complex—traits that suit it uniquely to the promise of AI. Yet the commercial pipeline of AI tools has been slow and often biased toward common diagnostic entities with large market potential. For rare diseases, pediatric subtypes, or region‐specific pathologies, the incentives for commercial development are scant.

The article by Calderaro *et al* [2] argues that much like in‐house laboratory‐developed tests such as special stains and immunohistochemistry, homebrew AI algorithms should undergo validation and be incorporated into the digital pathology workflow, which will ultimately enhance and grow the field of pathology and benefit more patients. Homebrew AI, under local pathologist oversight, offers a path forward that is agile, inclusive, and clinically grounded. However, in certain countries, including the United States, regulatory barriers may hinder their adoption and limit the potential benefits of these tools. This is particularly important, as board‐certified practicing pathologists should have the ability to directly develop and oversee validated algorithms, especially for rare diseases, thereby ensuring their safe and effective use, rather than leaving this responsibility solely to federal agencies such as the U.S. Food and Drug Administration (FDA).

## Development & validation

Homebrew AI models refer to algorithms that are developed and validated within local laboratories and are primarily used for immunohistochemical stain (IHC) quantification, and diagnostic and prognostic predictions [3]. Prior to deployment and similar to traditional laboratory diagnostics, AI algorithms for use in digital pathology pass through several milestones. These milestones include hypothesis formulation, preprocessing and annotations of training data, designing and training the model, and clinical validation [4]. In the United States, the American College of Pathologists (CAP) sets guidelines for validating AI algorithms in digital pathology [5]. Indeed, CAP state that prior to utilizing an AI algorithm in practice, such systems must be validated along with revalidation of a prior system in case of changes within the algorithm or model. Further, the validation must be done on a minimum number of 60 whole‐slide‐images (WSI). Moreover, the use of non‐FDA approved systems must be clearly stated on the pathology report.

## Implementation & sustainability

Calderaro *et al* articulate a compelling rationale for enabling local, noncommercial AI model development and deployment in pathology departments. Drawing parallels with the long‐standing practice of laboratory‐developed tests (LDTs), the authors advocate for analogous validation frameworks for AI‐enabled tools. They make a persuasive case that homebrew AI offers a route to innovation that bypasses the stifling pace, cost, and rigidity of commercial FDA *in vitro* diagnostic (IVD) device pathways. According to the authors, implementing noncommercial homebrew AI algorithms may well improve the diagnostic workflow in digital pathology by enhancing IHC interpretation and increasing the accuracy of pathology reports.

Local laboratories should implement quality control procedures for homebrew AI algorithms comparable to those used for conventional laboratory tests. Such procedures include assessing appropriate positive and negative controls, software calibration, keeping with maintenance logs, and regular personnel training. Certainly, the laboratory director should be responsible for conducting periodic inspections and quality assurance of the developed homebrew AI models [5]. Integrating homebrew AI algorithms into pathology residency training and clinical laboratories is urgently called for, but faces several barriers, including limited foundational skills in computational pathology and a shortage of qualified experts [2, 6], raising concerns about widespread implementation.

## Opportunities

Several opportunities and strengths of homebrew AI models may include:

- Flexibility for Local Contexts: AI models trained and validated in local laboratories can be fine‐tuned to institutional staining techniques, scanner settings, and patient demographics, which improves performance and reliability.
- Equity in Access: By circumventing commercial bottlenecks, homebrew models have the potential to offer underserved patient groups access to cutting‐edge tools.
- Expert Oversight: Embedding AI development under the guidance of expert pathologists strengthens both interpretability and accountability.
- Regulatory Compatibility: The authors demonstrate that current EU provisions (IVDR Article 5 [5]) and, to a more limited extent, CLIA rules in the US, permit such in‐house innovations under specific conditions.

This approach is practical and resonates with the lived experience of practicing pathologists who routinely interpret semiquantitative IHC assays such as Ki‐67 or HER2. Applying similar oversight to AI tools is a natural progression.

## Real‐world barriers for practicing pathologists

Despite the potential opportunities and strengths of their argument, significant limitations, barriers, and operational gaps must be addressed to transform this vision into sustainable practice:

- Infrastructure Gaps: Many pathology departments, particularly in publicly funded health systems or resource‐constrained regions, still lack essential components like high‐resolution slide scanners, robust data storage, or secure cloud computation frameworks.
- Workforce and Training Deficits: As Calderaro *et al* [2] acknowledge, AI literacy is not yet embedded in most residency or continuing professional development programs.
- Legal and Insurance Ambiguity: When a homebrew model contributes to a diagnostic error, responsibility may rest solely with the pathologist. The absence of clear legal precedents around AI‐driven diagnostic decision‐making raises malpractice concerns.
- Quality Control Complexity: Unlike traditional IHC markers with discrete endpoints, many AI models generate probabilistic heatmaps or gradient overlays. Developing robust QC pipelines that account for interpretability, reproducibility, and calibration remains technically challenging.
- Sustainability of Oversight: Homebrew models require ongoing monitoring for ‘model drift,’ and periodic retraining. For overstretched pathology services already grappling with staffing shortages, this added burden could limit uptake.

## Conclusion

Calderaro *et al* are commended for reframing the AI‐in‐pathology debate around the agency of the practicing pathologist. By placing trust in the profession and recognizing the existing culture of laboratory‐developed testing, they offer a future of AI that is human‐led, not corporate‐controlled. Yet this enthusiasm must be tempered with realism, considering the aforementioned limitations and barriers to implementation. Without coordinated national efforts, sustained investment in digital infrastructure, and legal reform, the promise of homebrew AI may remain confined to elite academic centers or a select few first‐to‐innovate laboratories.

## Author contributions statement

Both authors conceptualized, wrote, and revised this article.

## Data Availability

Data sharing not applicable to this article, as no datasets were generated or analyzed during the current study.

## References

[1] Snead DR , Azam AS , Thirlwall J , *et al*. Variation within and between digital pathology and light microscopy for the diagnosis of histopathology slides: blinded crossover comparison study. Health Technol Assess 2025; 29: 1–75. 10.3310/SPLK4325 PMC12278374 40654002

[2] Calderaro J , Morement H , Penault‐Llorca F , *et al*. The case for homebrew AI in diagnostic pathology. J Pathol 2025; 266: 390–394. 40613320 10.1002/path.6438 PMC12256376

[3] Poalelungi DG , Neagu AI , Fulga A , *et al*. Revolutionizing pathology with artificial intelligence: innovations in immunohistochemistry. J Pers Med 2024; 14: 693. 39063947 10.3390/jpm14070693 PMC11278211

[4] Makhlouf Y , Salto‐Tellez M , James J , *et al*. General roadmap and core steps for the development of AI tools in digital pathology. Diagnostics (Basel) 2022; 12: 1272. 35626427 10.3390/diagnostics12051272 PMC9141041

[5] How to Validate AI Algorithms in Anatomic Pathology [Internet] . American College of Pathologists, [Accessed August 20, 2025]: Available from: https://www.cap.org/member-resources/clinical-informatics-resources/how-to-validate-ai-algorithms-in-anatomic-pathology.

[6] Vos S , Hebeda K , Milota M , *et al*. Making pathologists ready for the new artificial intelligence era: changes in required competencies. Mod Pathol 2025; 38: 100657. 39542175 10.1016/j.modpat.2024.100657

